# Fecal Calprotectin in Self-Reported Milk Intolerance: Not Only Lactose Intolerance

**DOI:** 10.3390/nu15041048

**Published:** 2023-02-20

**Authors:** Aurelio Seidita, Pasquale Mansueto, Alessandra Giuliano, Marta Chiavetta, Maurizio Soresi, Antonio Carroccio, the Internal Medicine Study Group

**Affiliations:** 1 Unit of Internal Medicine, “V. Cervello” Hospital, Ospedali Riuniti “Villa Sofia-Cervello”, 90120 Palermo, Italy; 2 Department of Health Promotion Sciences, Maternal and Infant Care, Internal Medicine and Medical Specialties (PROMISE), University of Palermo, 90127 Palermo, Italy; 3 Unit of Internal Medicine, Department of Health Promotion Sciences, Maternal and Infant Care, Internal Medicine and Medical Specialties (PROMISE), University of Palermo, 90127 Palermo, Italy

**Keywords:** milk intolerance, lactose intolerance, fecal calprotectin, cow’s milk proteins allergy, LHBT, lactose hydrogen breath test, SRMI

## Abstract

The hypothesis is that inflammatory/allergic conditions should be considered in self-reported milk intolerance (SRMI) patients who test negative and/or are asymptomatic at Lactose Hydrogen Breath Test (LHBT). We analyzed fecal calprotectin (FCP) values in SRMI patients to investigate the frequency of a “positive” intestinal inflammation marker and its correlation with lactose tolerance/intolerance. Data from 329 SRMI patients were retrospectively analyzed; according to the positive/negative results (maldigester/digester) and the presence/absence of symptoms reported during LHBT (intolerant/tolerant), patients were divided into: ‘lactose tolerants’ (n. 104), ‘maldigesters/intolerants’ (n. 187), ‘digesters/intolerants’ (n. 38). FCP values were analyzed in all three subgroups. A percentage of SRMI patients complained of constipation (>15%), extraintestinal symptoms (>30% including anemia), multiple food hypersensitivity (7.6%) and had intraepithelial lymphocytic infiltration at duodenal biopsy (>50%). Over 50.0% showed FCP values above the normal limit. Lactose tolerants and maldigesters/intolerants had higher positivity frequencies (*p* < 0.0001, for both) and absolute values (*p* = 0.04, for maldigesters/intolerants) of FCP compared to digesters/intolerants. FCP was not useful to differentiate tolerant from intolerant subjects (AUC 0.58). Our data suggest the existence of an allergic/inflammatory pathogenetic mechanism in a subset of SRMI subjects. FCP results are in keeping with this hypothesis, even if they cannot differentiate lactose tolerant from intolerant patients.

## 1. Introduction

Lactose intolerance is the most frequent food intolerance worldwide. It is a condition in which the intake of cow’s milk (CM) and fresh dairy products causes a non-immune-mediated reaction characterized by gastrointestinal (mainly diarrhea, abdominal pain, bloating, acid regurgitation, retrosternal heartburn, nausea and dyspepsia) and, to a lesser extent, extraintestinal symptoms (especially fatigue, skin disorders and headache) [1,2].

The pathogenesis of the disease is attributable to a deficiency of lactase, the enzyme responsible for the digestion of lactose. Lactose is the sugar contained in CM and its fresh derivatives; it is not present in aged ones thanks to the bacterial digestion processes typical of aging. In the presence of a congenital or acquired (“secondary”) lactase deficiency, lactose cannot be digested and it accumulates in the gut, where—being osmotically active—it leads to an increase in the influx of liquids; this effect, associated with its fermentation by the local microbial flora, gives rise to the typical symptoms of lactose intolerance. If lactose intolerance is suspected, this condition can be easily diagnosed by the Lactose Hydrogen Breath Test (LHBT) [3]. This test measures the concentration of hydrogen (H_2_) in the air exhaled by the patient after the oral consumption of lactose, produced by the intestinal fermentation of the sugar [4,5]. However, many patients with self-reported milk intolerance (SRMI) test negative and/or are asymptomatic after the LHBT lactose load. In these subjects, an alternative diagnosis should be considered.

In fact, CM can also cause disorders through immune-mediated mechanisms, specifically directed against CM proteins (CM protein allergy, CMPA). CMPA is widespread, especially among children. Although all CM proteins can act as possible allergens, casein and some whey proteins [in particular beta-lactoglobulin (BLG), alpha-lactalbumin (ALA), bovine serum albumin (BSA), lactoferrin and immunoglobulins] are believed to be responsible for most allergic sensitizations. While in children CMPA is often caused by an immunoglobulin E (IgE)-mediated reaction detectable by a skin prick test (SPT) or specific IgE assay (e.g., RadioAllergoSorbent Test, RAST), in adults it has been hypothesized that immune activation may not occur via an IgE-mediated pathway, but could be due to other mechanisms, probably cell-mediated, that cannot be demonstrated with existing common laboratory tests. For this reason, CMPA diagnosis in adults is mainly clinical and based on patient history: the presence of symptoms is caused by the intake of CM and its derivatives, their disappearance in a CM-free diet and their reappearance in a double-blind placebo-controlled (DBPC) CM-protein challenge [6].

Because of this complex diagnostic process, in the absence of a specific diagnostic biomarker, CMPA diagnosis is almost never taken into consideration in SRMI subjects, and instead, a diagnosis of lactose intolerance or irritable bowel syndrome (IBS) is “automatically” made [7,8].

Recently, we demonstrated that a large percentage of SRMI adult patients were not suffering from lactose intolerance. They tested negative on LHBT, but a double-blind placebo-controlled (DBPC) milk-protein challenge revealed clinical reactions after CM intake [8]. Some studies have shown the presence of slightly elevated fecal calprotectin (FCP) values in patients with food allergy, especially in pediatric IgE-mediated CM allergy [9,10,11].

Thus, in SRMI patients, the hypothesis of an underlying inflammatory/allergic condition should be considered. However, no previous studies have evaluated the behavior of FCP in SRMI subjects.

The aim of our retrospective study was to analyze FCP values in adult patients with SRMI, subdivided according to their LHBT clinical response, to investigate: (A) the frequency of a “positive” marker of intestinal mucosa inflammation; (B) the possibility of differentiating subjects suffering from “true” lactose intolerance from those who did not have a clinical reaction to LHBT.

## 2. Materials and Methods

We retrospectively included patients with SRMI referred to three Hub Centers of Celiac Disease and Food Intolerance in Italy [Department of Internal Medicine, University Hospital of Palermo; Department of Internal Medicine, “V. Cervello” Hospital, Palermo; Department of Internal Medicine, Hospital of Sciacca (Agrigento)], between January 2017 and June 2020.

The following patient data were recorded: demographic characteristics, clinical presentation, associated autoimmune diseases, presence of other food allergies/intolerances [Non-Celiac Wheat Sensitivity (NCWS), Systemic Nickel Allergy Syndrome (SNAS), and Multiple Food Hypersensitivity (MFH)], type of foods causing symptoms (fresh CM and its fresh and aged derivatives), LHBT results [both in terms of H_2_ excretion and appearance of clinical symptoms both during and within 24 h after the test], SPT and/or RAST for CM proteins, and FCP values before diagnosis on a diet containing CM and its derivatives. In a subgroup of patients, the presence of Human Leukocyte Antigens (HLA) DQ2 and DQ8 and duodenal mucosal lesions, such as increased number of intraepithelial lymphocytes, crypt hyperplasia, and different degrees of villous atrophy, described according to the Marsh–Oberhuber classification were also recorded [12,13].

Patients started a CM and its derivatives-free diet only after the execution of tests, such as LHBT, SPT and/or RAST for CM proteins and FCP.

### 2.1. Inclusion Criteria of SRMI Patients

All SRMI patients included in the study met the following requirements: (a) age between 18 and 65 years; (b) SRMI, with the consequent exclusion of CM and its derivatives, both fresh and aged from the diet for at least one year; (c) complete remission of gastrointestinal and extraintestinal symptoms strictly associated with the intake of CM and its derivatives, on a diet without these foods; (d) reported the reappearance of symptoms on all occasions in which CM and its derivatives were consumed, whether voluntarily or accidentally; (e) follow-up of at least one year after the LHBT; (f) at least two follow-up visits.

### 2.2. Exclusion Criteria of SRMI Patients

All cases with (a) age < 18 and >65 years; (b) incomplete medical records; (c) other previously diagnosed “organic” gastrointestinal diseases; (d) ongoing gastrointestinal or systemic infections; (e) treatment with antibiotics, prebiotics, probiotics, immunosuppressants and corticosteroids, (f) ongoing CM free diet at the first visit.

### 2.3. LHBT

The LHBT was performed after a 12-h overnight fast. All subjects were given a standard dose of 25 g of lactose, suspended in 250–300 mL of water (0.30 moles per liter). A basal alveolar breath sample was taken prior to lactose ingestion. Subsequent alveolar breath samples were collected at 30-min intervals over a 3-h period following ingestion and the H_2_ concentration in each breath sample was measured using the Quintron Microlyzer (Spectra 2000 Srl., Rome, Italy). Subjects were instructed not to eat or drink anything until the test was completed. All tested subjects were questioned during the test and contacted within 24 h after the test to assess the possible reappearance of gastrointestinal or extraintestinal symptoms after the challenge.

In patients with SRMI but negative for LHBT, to rule out a false negative result due to the presence of non-H_2_-producing microbiota, a lactulose breath test with 12 g of suspended lactulose in 120 mL of water was administered in the days following the LHBT. Patients negative at the LHBT and lactulose breath test were excluded from the study because they did not possess H_2_-producing flora.

Patients with H_2_ concentrations in alveolar breath samples > 20 ppm over baseline were defined as ‘maldigesters’, while those with lower concentrations were defined as ‘digesters’.

We also defined ‘lactose intolerants’ as those patients with one or more symptoms during and within 24 h after the test, and ‘lactose tolerants’ were those without symptoms.

Consequently, the statistical analysis considered three subgroups of SRMI patients:

- ‘lactose tolerants’ (maldigesters/tolerants plus digesters/tolerants);
- ‘maldigesters/intolerants’;
- ‘digesters/intolerants’.

**Figure 1:**
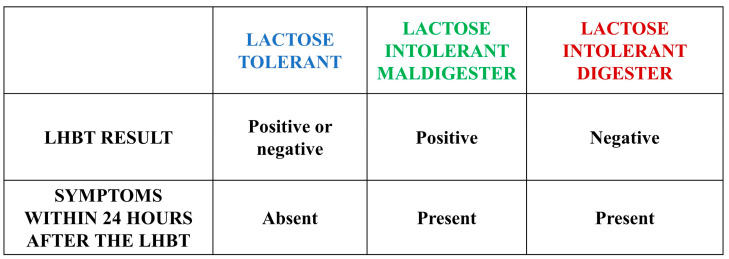
Characteristics of the three subgroups.

A summary is shown in Figure 1.

### 2.4. Fecal Calprotectin

#### 2.4.1. Collection of the Fecal Samples

To obtain the fecal samples, during a diet containing CM and its derivatives, the patients collected a small amount of stool with a collection scoop and placed it in a sterile container, without any preservatives. The sample was stored in the patient’s refrigerator and delivered to the laboratory within 24 h after collection.

#### 2.4.2. FCP Measurement

FCP was measured with a commercially available quantitative enzyme immunosorbent assay (Calprest, Eurospital, Trieste, Italy). After collecting about 100 mg of feces (range 40–120 mg), the extraction solution, diluted in a weight-volume ratio of 1:50, was added to the sample. After vigorous vortexing for about 30 s, the sample was homogenized for about 25 min. Then, the homogenate (1 mL) was centrifuged for 20 min at room temperature. Subsequently, 0.5 mL of the supernatant was further diluted 1:50 with the dilution buffer and placed in duplicate in the wells of the plate, according to the manufacturer’s instructions. After two cycles, each including an incubation of about 45 min and three washing operations, 100 µL of the enzyme-substrate was added to each well and, after a further incubation at room temperature, a biologist read the optical density (absorbance) at a wavelength of 405 nm. The results were calculated using the manufacturer’s instructions and the calprotectin concentration of each sample was expressed in µg/g, with values below 50 being the norm.

### 2.5. Statistical Analysis

Data were expressed as mean ± standard deviation (SD) when the distribution was Gaussian and Student’s t-test was used to evaluate differences between the groups. Comparisons between more than two groups were performed with ANOVA, followed by a posthoc analysis using the Bonferroni test. Otherwise, data were expressed as the median and interquartile range (IQR) and analyzed with the Mann–Whitney U test. The χ^2^ test and Fisher’s exact test were used to compare frequency values across population groups.

All subjects agreed to participate in the study. The study was approved by the Ethics Committee of the University Hospital of Palermo (n. 10/2019) and registered on the ClinicalTrials.gov website (protocol n. NCT03008252).

## 3. Results

**Figure 2:**
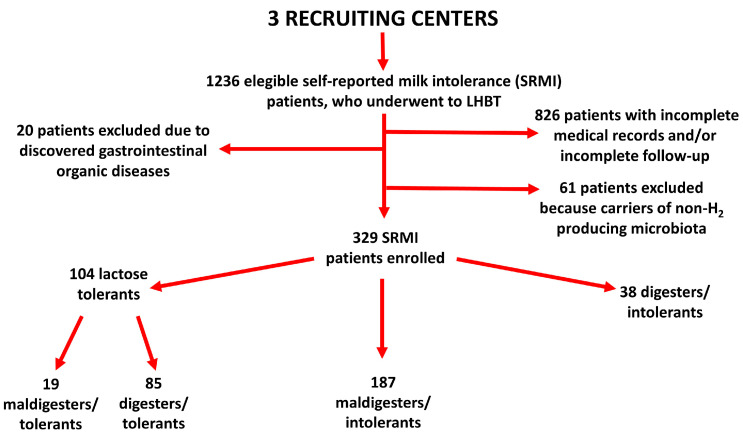
Flow-chart of the study, showing distribution of the patients in different subgroups according to LHBT results and appearance of symptoms after LHBT test.

During the study period, 1236 patients with SRMI underwent LHBT. After the application of the inclusion/exclusion criteria, 329 patients were eligible and then included in the study population. The lactose-tolerant subgroup was composed of 104 patients (19 maldigesters/tolerants and 85 digesters/tolerants), the maldigesters/intolerant subgroup 187 patients, and the digesters/intolerants subgroup 38 patients (Figure 2).

Table 1 shows the demographic, clinical and histological characteristics of the general population of SRMI patients and of the three subgroups.

Most of the patients with SRMI were female (82.4%), aged between the third and fourth decade of life and complained of both gastrointestinal symptoms—IBS-like (67.9%) and functional dyspepsia-like (74.2%)—and extraintestinal symptoms (22.8%), anemia (18.8%), and weight loss (19.8%) (for further details, see Table S1 in the Supplementary File). This was often associated with NCWS (31.9%), the presence of HLA DQ2/DQ8 (53.5%), and duodenal intraepithelial lymphocytosis (Marsh 1, 51.2%, data available in 80 subjects). No patients proved positive for SPT and/or RAST to CM proteins. Statistically significant differences were demonstrated between lactose tolerants and maldigesters/intolerants regarding the female sex (*p* = 0.02) and the presence of anemia (*p* = 0.02) and MFH (*p* < 0.04). In addition, statistically significant differences were found between lactose tolerants and digesters/intolerants regarding the age at symptoms onset (*p* = 0.03). Finally, statistically significant differences were found between maldigesters/intolerants and digesters/intolerants regarding the age at symptom onset (*p* < 0.02), and the presence of MFH (*p* < 0.02). We did not demonstrate any other statistically significant differences between the other variables examined, including duodenal histology.

Table S2 (see the Supplementary File) shows the reported milk products not tolerated both by the entire SRMI population and the three subgroups. Table 2 shows the respective FCP values before the start of the elimination diet excluding milk and its derivatives, both fresh and aged.

We demonstrated a statistically significant higher frequency of FCP positivity (i.e., >50 mcg/g) among lactose tolerants and maldigesters/intolerants compared to digesters/intolerants (*p* < 0.0001, for both). Similarly, in the context of patients with positive FCP, we demonstrated statistically significant higher absolute FCP values among lactose tolerants than in digesters/intolerants (*p* = 0.04).

**Figure 3:**
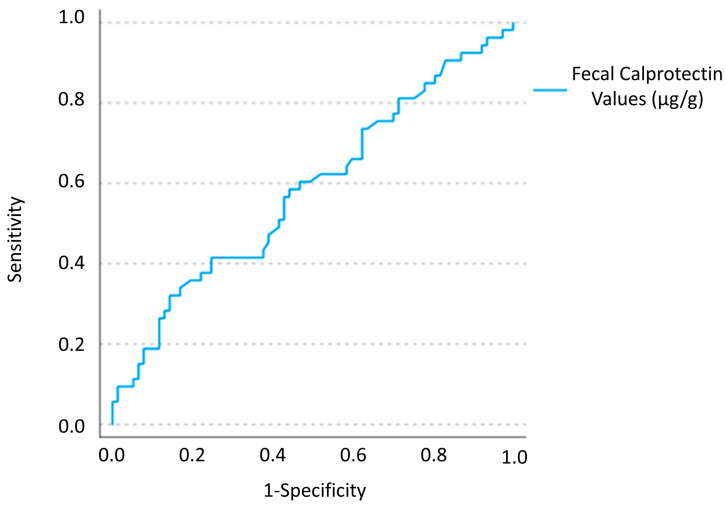
Receiver operating characteristic (ROC) curve analysis of the FCP values in the lactose tolerants vs. all intolerants.

Finally, Figure 3 shows the results of the receiver operating characteristic (ROC) curve analysis of the FCP values in the lactose tolerants vs. all intolerants, giving an area under the curve (AUC) of 0.58 (95% confidence interval 0.46–0.65).

## 4. Discussion

Lactose intolerance is the most frequent food intolerance in the world. Its prevalence is estimated to be around 68% of the global population. However, this very high percentage does not correspond to an identical frequency of “true” lactose maldigestion due to lactase deficiency. It is known that other factors, in addition to lactase deficiency, might contribute to the onset of symptoms after lactose ingestion in subjects with lactose maldigestion. Among these, a concomitant condition of IBS, probably secondary to visceral hypersensitivity, might characterize this specific disease. Consequently, SRMI is very common in patients with IBS, and lactose intolerance is often diagnosed in these patients regardless of the LHBT results [3,5,14,15].

On the other hand, little is known about those patients who report symptoms after consuming CM and dairy products, including aged ones (therefore, lactose-free), and are asymptomatic after LHBT, regardless of the test results (i.e., lactose-tolerant patients) [8].

The lack of correspondence between the self-reported symptoms after intake of CM and its derivatives and LHBT results have already been reported in the literature [5,16,17,18,19,20,21] with a wide percentage range (4–86%) of LHBT positivity in patients with SRMI [22].

Many explanations could be proposed for this lack of correlation. In the case of lactose tolerants, the symptoms are not caused by the ingestion of lactose but could result from an abnormal response to other components of the milk, such as proteins. A pathogenetic hypothesis could be the presence of a concomitant underlying allergic/inflammatory process, as in the case of CMPA. However, CMPA is difficult to diagnose in adults because of the absence of biochemical markers. In fact, CMPA in adults may be caused by an allergic/inflammatory, non-IgE-mediated mechanism, which seems to be cell-mediated. A possible inflammatory marker could be FCP, a calcium-binding protein, found mostly in neutrophils and macrophages. It has a regulatory function in the inflammatory processes, with antimicrobial and antiproliferative activities. FCP is a marker of inflammation in the gastrointestinal tract, and its presence in feces is probably due to the migration of myeloid cells into the intestinal mucosa [23]. Among the neutrophil-derived inflammatory markers, FCP is preferred thanks to the non-invasiveness of the test and its stability in feces and at room temperature for over 7 days [9,24,25]. FCP is used to differentiate inflammatory bowel disease (IBD) or non-IBD inflammatory intestinal disease (i.e., infective and inflammatory intestinal disorders and malignancies) from intestinal functional diseases, such as IBS. Several studies have highlighted its diagnostic, prognostic, and monitoring role in IBD [10,26]. In fact, in IBD patients, the chronic inflammation of the intestinal wall and/or mucosa is responsible for structural gut damage, highlightable on diagnostic exams [27]. On the other hand, IBS is a functional, non-inflammatory, gastrointestinal disorder, defined according to the Rome IV criteria. Motility disturbance, visceral hypersensitivity, altered mucosal and immune function, altered gut microbiota, altered central nervous system processing and the absence of signs of disease at the imaging or at colonoscopy are its main features [28].

Although many studies have demonstrated that FCP values can distinguish between IBD and IBS, others have shown that the presence of low-grade inflammation in IBS, a disease with a multifactorial etiology, can also lead to a slight increase in FCP in these patients. Further research is needed to understand whether increased FCP values in IBS patients could be useful to select patients who would benefit from anti-inflammatory therapy [29]. Other studies have demonstrated elevated values of FCP in active celiac disease [30,31]; more severe villous atrophy and increased intestinal permeability appear to be the causes of the increased values of FCP in these patients [23].

FCP positivity has also been observed in allergic pathologies, such as allergic colitis [9], food protein-induced enterocolitis syndrome [32], IgE and non-IgE mediated CMPA and multiple food allergies in children. Moreover, a decrease in FCP values after the elimination of the trigger food(s) from the diet was noted [10,33,34]. In addition, some recent studies have shown the putative diagnostic and predictive therapy-response role of FCP in children with CMPA [35,36].

Little is known about FCP in SRMI adult patients who could also be suffering from CMPA. Thus, the aim of our study was to assess FCP values in SRMI adult patients divided into different groups according to the assay result and the onset of symptoms after LHBT.

In our study, we included a large group of adult patients with SRMI, rigorously selected in third-level centers for the diagnosis and therapy of food allergies and intolerances and functional gastrointestinal disorders. All the enrolled SRMI patients’ self-reported symptoms (gastrointestinal and/or extraintestinal) were strictly associated with the intake of CM and its derivatives, both fresh and aged.

According to the LHBT results, only 56.9% of the entire group of SRMI were maldigesters/intolerants. Of the remaining patients, 31.6% were lactose tolerants, and 11.5% were digesters/intolerants. More than 50% of the whole SRMI group showed FCP values above the normal limit, with a statistically significant higher frequency and higher absolute FCP values in lactose tolerants and maldigesters/intolerants compared to digesters/intolerants. This latter group could have been responding psychologically to a nocebo effect, and consequently could be considered as a not-inflamed control group. Our data could lead us to suppose that an inflammatory process may be present especially in lactose tolerants, but also in maldigesters/intolerants. In addition to the FCP positivity, other features seem to corroborate the inflammatory hypothesis and the probable presence of CMPA in our SRMI patients, especially in the lactose tolerants. Clinically, an inflammatory mechanism could be related to the presence of some symptoms which were present in about 20% of the entire SRMI group: constipation (isolated or in mixed bowel habits), extraintestinal symptoms, and anemia. Constipation, in particular, cannot be considered a consequence of lactose intolerance, by definition characterized by osmotic diarrhea, while it has been described as one of the symptoms of CMPA, both in pediatric [37,38,39,40] and in adult patients [41,42]. Furthermore, the extraintestinal symptoms reported by our SRMI patients remain difficult to explain if a “simple” lactose intolerance were the case [14]. Finally, anemia, which was more frequent in the subgroup of lactose tolerants compared to maldigesters/intolerants, on the one hand, could be linked to strict dietary self-limitation that can lead to malnutrition, but on the other hand, concomitant and subtle intestinal malabsorption due to chronic inflammation cannot be excluded. In this regard, a possible role for the “allergic/inflammatory” hypothesis for at least a part of our SRMI patients, particularly for the lactose tolerants, may also be suggested by the high frequency of DQ2/DQ8 haplotypes (53.5%), as well as by their duodenal histological data (Marsh 1 in 51.2%). Haplotypes DQ2 and DQ8 are expressed by antigen-presenting cells and have high binding affinity to deamidated gliadin peptides, thereby inducing a T-cell-mediated inflammatory reaction in the small intestinal mucosa, which results in genetic susceptibility to the development of celiac disease [43]. Our data on HLA and lymphocyte infiltration of the duodenal mucosa could suggest that a percentage of patients with SRMI may be suffering from CMPA, with a pathogenetic model similar to that proposed for NCWS patients [44,45].

Furthermore, the associated NCWS observed in one-third of our SRMI patients is in line with our previous experience of a frequent association between CMPA and NCWS [44,46,47] and with the results of previous confocal endomicroscopy studies in IBS patients, where most of the subjects studied showed a multiple food “reaction” (not only to milk, but also to wheat, and other foods) [45,48].

Moreover, in our study group, we observed a higher frequency of MFH in lactose tolerants and digesters/intolerants compared to maldigesters/intolerants (*p* < 0.04 and *p* < 0.02, respectively). In lactose tolerants, these data could be explained, at least in part, by the putative presence of an allergic/inflammatory mechanism based on a complex disorder of antigen presentation, which could support the association between CMPA and other food allergies. On the contrary, in the digesters/intolerants group, the association with IBS could explain the high frequency of a multiple food “reaction”.

These clinical, genetic and histological data, together with the high positivity rate of FCP in our SRMI patients might suggest the existence of a pathogenetic mechanism against CM proteins different from that of a “simple” enzymatic deficiency. The subgroup of lactose tolerants, who had the highest frequency and absolute values of FCP positivity, might be characterized by a higher inflammatory status due to a higher prevalence of patients with CMPA, whereas digesters/intolerants could be suffering especially from IBS. In this latter group, in fact, symptoms could be caused by a simple “nocebo effect” or by trigger effect on visceral hypersensitivity when patients consume milk and/or dairy products, or by the concomitant consumption of other foods rich in other Fermentable, Oligo-, Di-, Monosaccharides and Polyols (FODMAPs), known for their ability to trigger symptoms in patients with IBS [3,14]. By contrast, the maldigesters/intolerants might have a “true” lactose intolerance, but in a percentage of cases, this could probably be associated with a slight intestinal mucosa inflammation. This inflammatory substrate could explain the high frequency of FCP positivity (51.9%) also in this subgroup. On this basis, the ROC curve analysis was not useful to differentiate between lactose tolerants (hypothetically the “more inflamed”) and lactose intolerants. In addition, it is interesting to note that 25.7% of the maldigesters/intolerants self-reported symptoms after ingesting aged milk derivatives (e.g., parmesan), which are by definition lactose-free. This would confirm the possible coexistence of SRMI patients suffering from both lactose intolerance and CMPA.

Some limitations of our study must be underlined. Primarily, our study was carried out in three tertiary centers with experience in the field of food-related allergic diseases; consequently, it is possible that we observed a preselected population, composed of patients who do not show a “simple” lactose intolerance, as often observed in the general population. Another selection bias could be related to the high frequency of female patients. According to a recent review of the literature, not only have several authors identified a higher incidence of “lactase non-persistence” among women, the elderly and some ethnic groups, but also a higher perception of lactose intolerance (and, consequently, the adoption of a dairy-free diet) among women has been highlighted in the last decades. Nonetheless, the review authors conclude that there is insufficient evidence to support a direct association between lactose intolerance and gender, age, or ethnicity [49]. Other limits are the high frequency of DQ2/DQ8 haplotypes and the high percentage of a coexisting diagnosis of NCWS, a condition that some authors report as being associated with the activation of both the innate and acquired immune systems. Furthermore, we are aware that FCP is mainly a marker of neutrophil activation, thus one of the main limits of our study is the lack of an analysis of specific eosinophil and mastocyte activation markers. Finally, although the presence of histological alterations in the rectum is well known, especially in the pediatric CMPA population [50], this investigation was not carried out in our adult population.

The strengths of our study were the high number of patients included, the standardized method of performing LHBT in just three centers and the strict inclusion criteria. Finally, to our knowledge, our study is the first to have evaluated FCP values in the context of adult SRMI, lactose intolerance, and suspected CMPA.

## 5. Conclusions

In our study, only one-third of SRMI patients were actually diagnosed with lactose intolerance by LHBT. Clinical data (high prevalence in females, presentation with constipation in more than 15% of patients, and high frequency of extraintestinal symptoms, NCWS and MFH), laboratory data (>50% of patients positive for HLA-DQ2/DQ8), and biopsy findings (intraepithelial lymphocytic infiltration in about 50% of patients) seem to suggest the existence of a different pathogenetic mechanism, probably of an allergic/inflammatory nature, rather than a “simple” enzyme deficiency. The FCP results are in keeping with this hypothesis, being positive in over 50% of SRMI patients, and with the evidence that about one-third of these patients reported the onset of symptoms even after ingestion of aged CM derivatives, which seems to suggest that at least some of these patients may be suffering from CMPA. However, to confirm this hypothesis, a broad prospective investigation is required, specifically designed for the evaluation of the immunological/inflammatory hypothesis and, more specifically, of an allergic-like pathogenetic mechanism in the SRMI context.

From a practical point of view our data suggest that among SRMI patients, there are two different main groups. One consists of the “typical” lactose maldigester/intolerant patients, and the other one of the lactose tolerant patients, who probably suffer from CMPA.

We believe that the patients who find a correlation between symptoms onset and dairy intake should undergo a non-invasive, inexpensive test, such as the LHBT. In the event of a positive LHBT associated with clinical symptoms (positive test), the physicians can suggest a diet containing lactose-free foods and make patients aware of the availability of different pharmaceutical forms of lactase, to prevent the appearance of symptoms after lactose intake. Moreover, these patients (maldigester/intolerant) have their own threshold of tolerability for lactose-containing foods, so a strict lactose-free diet is not necessary, and a reduction in the amount of ingested lactose-containing foods, based on individual tolerability, can be suggested.

In our opinion, a different approach should be considered in symptomatic patients with negative LHBT (i.e., “lactose tolerant”), assuming that these patients are probably sensitive to other components of CM, maybe proteins, as in CMPA. In this case, the disease could be more severe than “a simple” lactose intolerance, so it is very important to follow a rigorous CM and derivatives-free diet.

Unfortunately, the data of the present study showed that FCP values do not seem useful in distinguishing between these two groups.

## Figures and Tables

**Table 1:** Demographic, clinical, and histological characteristics of patients with SRMI.

	SRMI ^1^ (n = 329) (%) (A + B + C)	SRMI ^1^ Lactose Tolerants ^2^ (n = 104) (%) (A)	SRMI ^1^ Maldigesters/ Intolerants ^3^ (n = 187) (%) (B)	SRMI ^1^ Digesters/ Intolerants ^4^ (n = 38) (%) (C)	*p*
Gender					A vs. B 0.02
Female	271 (82.4)	78 (75.0)	162 (86.6)	31 (81.6)	
Male	58 (17.6)	26 (25.0)	25 (13.4)	7 (18.4)	
Age at symptoms onset (years) (mean ± SD ^10^)	28.8 ± 13.9	29.5 ± 14.1	29.4 ± 14.0	22.5 ± 10.6	A vs. C 0.03 B vs. C < 0.02
IBS ^5^-like symptoms					
None	106 (32.2)	30 (28.8)	67 (35.8)	9 (23.7)	NS ^11^
Diarrhea	168 (51.1)	55 (52.9)	92 (49.2)	21 (55.3)	NS ^11^
Constipation	13 (4.0)	6 (5.8)	5 (2.7)	2 (5.3)	NS ^11^
Mixed bowel habits	42 (12.8)	13 (12.5)	22 (12.3)	6 (15.8)	NS ^11^
Dyspepsia-like	244 (74.2)	74 (71.2)	143 (76.5)	27 (71.1)	NS ^11^
Extraintestinal symptoms	75 (22.8)	31 (29.8)	38 (20.3)	6 (15.8)	NS ^11^
Anemia	62 (18.8)	28 (26.9)	28 (15.0)	6 (15.8)	A vs. B 0.02
Weight loss	65 (19.8)	25 (24.0)	34 (18.2)	6 (15.8)	NS ^11^
Autoimmune diseases	48 (14.6)	16 (15.4)	28 (15.0)	4 (10.5)	NS ^11^
Hashimoto thyroiditis	35 (10.6)	12 (11.5)	21 (11.2)	2 (5.3)	NS ^11^
Celiac disease	32 (9.7)	12 (11.5)	19 (10.2)	1 (2.6)	NS ^11^
NCWS ^6^	105 (31.9)	32 (30.8)	61 (32.6)	12 (31.6)	NS ^11^
SNAS ^7^	41 (12.5)	14 (13.5)	26 (13.9)	1 (2.6)	NS ^11^
MFH ^8^	25 (7.6)	12 (11.5)	7 (3.7)	6 (15.8)	A vs. B < 0.04 B vs. C < 0.02
HLA ^9^ DQ2-DQ8 positivity	176 (53.5)	63 (60.6)	99 (52.9)	14 (36.8)	NS ^11^
Duodenal biopsy Marsh classification					
Stage 0	37/80 (46.3)	12/22 (54.5)	23/48 (47.9)	2/10 (20.0)	NS ^11^
Stage 1	41/80 (51.2)	10/22 (45.5)	24/48 (50.0)	7/10 (70.0)	NS ^11^
Stage 2	2/80 (2.5)	0/22 (0.0)	1/48 (2.1)	1/10 (10.0)	NS ^11^

^1^ SRMI = Self-Reported Milk Intolerance; ^2^ Lactose tolerants = Lactose Hydrogen Breath test positive or negative without symptoms within 24 h after the test; ^3^ Maldigesters/intolerants = Lactose Hydrogen Breath test positive with symptoms within 24 h after the test; ^4^ Digesters/intolerants = Lactose Hydrogen Breath test negative with symptoms within 24 h after the test; ^5^ IBS = Irritable Bowel Syndrome; ^6^ NCWS = Non-Celiac Wheat Sensitivity; ^7^ SNAS = Systemic Nickel Allergy Syndrome; ^8^ MFH = Multiple Food Hypersensitivity; ^9^ HLA = Human Leukocyte Antigens; ^10^ SD = Standard Deviation; ^11^ NS = not significant.

**Table 2:** Fecal calprotectin in patients with SRMI.

	SRMI ^1^ (n = 329) (A + B+C)	SRMI ^1^ Lactose Tolerants ^2^ (n = 104) (%) (A)	SRMI ^1^ Maldigesters/ Intolerants ^3^ (n = 187) (%) (B)	SRMI ^1^ Digesters/ Intolerants ^4^ (n = 38) (%) (C)	*p*
Fecal calprotectin					A vs. C <0.0001 B vs. C <0.0001 A vs. C 0.04
Positive (r.v. ^5^ > 50 mg/g	172 (52.3)	60 (57.7)	97 (51.9)	15 (39.4)	
Median (IQR ^6^)	106	130	100	98	
(mg/g) > 50	(75–171)	(94.75–220.5)	(69.6–164.25)	(100–349)	

^1^ SRMI = Self-Reported Milk Intolerance; ^2^ Lactose tolerant = Lactose Hydrogen Breath test positive or negative without symptoms within 24 h after the test; ^3^ Maldigesters/intolerants = Lactose Hydrogen Breath test positive with symptoms within 24 h after the test; ^4^ Digesters/intolerants = Lactose Hydrogen Breath test negative with symptoms within 24 h after the test. ^5^ r.v. = reference values; ^6^ IQR = interquartile range.

## Data Availability

The data presented in this study are available on request from the corresponding author. The data are not publicly available due to privacy restrictions.

## Supplementary Materials

The following supporting information can be downloaded at: https://www.mdpi.com/article/10.3390/nu15041048/s1, Table S1: Extraintestinal symptoms of the patients with SRMI, divided by system, in order of frequency; Table S2: Intolerance towards cow’s milk and derivatives in patients with SRMI.
