# Supplementary material for: Fecal Calprotectin in Self-Reported Milk Intolerance: Not Only Lactose Intolerance
Source: Nutrients. 2023 Feb 20;15(4):1048. doi: 10.3390/nu15041048 (PMC9962554; doi:10.3390/nu15041048)
Supplement: Supplementary file 1 [file nutrients-15-01048-s001.zip › nutrients-2195040-supplementary.pdf]

Table S1. Extraintestinal symptoms of the patients with SRMI, divided by system, in order of frequency.

Extraintestinal symptoms	SRMI[1] (n=329) (%) (A+B+C)	SRMI[1] Lactose tolerants[2] (n=104) (%) (A)	SRMI[1] Maldigesters/ Intolerants[3] (n=187) (%) (B)	SRMI[1] Digesters/ Intolerants[4] (n=38) (%) (C)	*P*
Constitutional symptoms	18 (5.5)	5 (4.8)	12 (6.4)	1 (2.6)	NS[5]
Fatigue	17 (5.2)	4 (3.8)	12 (6.4)	1 (2.6)	NS[5]
Chills	1 (0.3)	1 (1.0)	0 (0.0)	0 (0.0)	NS[5]
Skin disorders	18 (5.5)	6 (5.8)	11 (5.9)	1 (2.6)	NS[5]
Itching	10 (3.0)	3 (2.9)	6 (3.2)	1 (2.6)	NS[5]
Swelling under the skin	3 (0.9)	1 (1.0)	2 (1.1)	0 (0.0)	NS[5]
Erythema and/or eczema	2 (0.6)	1 (1.0)	1 (0.5)	0 (0.0)	NS[5]
Sweating	2 (0.6)	1 (1.0)	1 (0.5)	0 (0.0)	NS[5]
Alopecia	1 (0.3)	0 (0.0)	1 (0.5)	0 (0.0)	NS[5]
Urticaria-angioedema	1 (0.3)	1 (1.0)	0 (0.0)	0 (0.0)	NS[5]
Skeletal disorders	4 (1.2)	1 (1.0)	3 (1.6)	0 (0.0)	NS[5]
Myalgia	3 (0.9)	1 (1.0)	2 (1.1)	0 (0.0)	NS[5]
Arthralgia	1 (0.3)	0 (0.0)	1 (0.5)	0 (0.0)	NS[5]
Neurosensorial disorders	38 (11.6)	17 (16.3)	18 (9.6)	3 (7.9)	NS[5]
Headache	33 (10.0)	15 (14.4)	15 (8.0)	3 (7.9)	NS[5]
Anxiety	2 (0.6)	1 (1.0)	1 (0.5)	0 (0.0)	NS[5]
Visual disturbances	2 (0.6)	1 (1.0)	1 (0.5)	0 (0.0)	NS[5]
Tinnitus	1 (0.3)	0 (0.0)	1 (0.5)	0 (0.0)	NS[5]
Oral disorders	8 (2.4)	3 (2.9)	5 (2.7)	0 (0.0)	NS[5]
Dysphagia	4 (1.1)	2 (1.9)	2 (1.1)	0 (0.0)	NS[5]
Glossitis	2 (0.6)	0 (0.0)	2 (1.1)	0 (0.0)	NS[5]
Recurrent aphthous stomatitis	2 (0.6)	1 (1.0)	1 (0.5)	0 (0.0)	NS[5]
Xerostomia	1 (0.3)	0 (0.0)	1 (0.5)	0 (0.0)	NS[5]
Respiratory disorders	6 (1.8)	1 (1.0)	4 (2.1)	1 (2.6)	NS[5]
Dyspnea	5 (1.5)	0 (0.0)	4 (2.1)	1 (2.6)	NS[5]
Cough	1 (0.3)	0 (0.0)	0 (0.0)	1 (2.6)	NS[5]
Sneezing	1 (0.3)	1 (1.0)	0 (0.0)	0 (0.0)	NS[5]
Cardiovascular disorders (Palpitations, Tachycardia, Extrasystoles)	6 (1.8)	2 (1.9)	4 (2.1)	0 (0.0)	NS[5]
Urinary disorders	2 (0.6)	0 (0.0)	1 (0.5)	1 (2.6)	NS[5]
Incontinence	1 (0.3)	0 (0.0)	1 (0.5)	0 (0.0)	NS[5]
Recurrent cystitis	1 (0.3)	0 (0.0)	0 (0.0)	1 (2.6)	NS[5]

[1]SRMI= Self-Reported Milk Intolerance; [2]Lactose tolerants= Lactose Hydrogen Breath test positive or negative without symptoms within 24 hours after the test; [3]Maldigesters/intolerants= Lactose Hydrogen Breath test positive with symptoms within 24 hours after the test; [4]Digesters/intolerants= Lactose Hydrogen Breath test negative with symptoms within 24 hours after the test; [5]NS=not significant

Table S2. Milk and products responsible for symptoms in patients with SRMI.

	SRMI[1] (n=329) (A+B+C)	SRMI[1] Lactose tolerants[2] (n=104) (%) (A)	SRMI[1] Maldigesters/ Intolerants[3] (n=187) (%) (B)	SRMI[1] Digesters/ Intolerants[4] (n=38) (%) (C)	*P*
Cow's milk	324 (98.5)	102 (98.1)	185 (98.9)	37 (97.4)	NS[5]
Fresh dairy products	240 (72.9)	78 (75.0)	133 (71.1)	29 (76.3)	NS[5]
Aged dairy products	97 (29.5)	35 (33.7)	48 (25.7)	14 (36.8)	NS[5]

[1] SRMI = Self-Reported Milk Intolerance; [2] Lactose tolerants = Lactose Hydrogen Breath test positive or negative without symptoms within 24 hours after the test; [3] Maldigesters/intolerants= Lactose Hydrogen Breath test positive with symptoms within 24 hours after the test; [4] Digesters/intolerants= Lactose Hydrogen Breath test negative with symptoms within 24 hours after the test; [5] NS=not significant.
